# 

**Published:** 1888-11-03

**Authors:** 


### The Student's Column.

### ROYAL COLLEGE OF PHYSICIANS OF LONDON.

The following gentlemen having conformed to the bye-laws and regulations, and passed the required examinations, were, at a meeting of the college on the 25th ult., admitted Licentiates, viz. :—

Adamson, H. G., St. Bartholomew's.
Andrew, F. C., Manchester.
Barker, F., St. Thomas's.
Beaver, R. A., Liverpool.
Blaxall, F. R., University College.
Boycott, A. N., St. Thomas's.
Bray, H. A., King's College.
Briscoe, J. E., Leeds.
Broadway, S. A. W. E., Charing Cross.
Burland, H , Manchester.
Cahill, W. A., Guy's.
Cant, F., Manchester.
Cheale, M., St. Bartholomew's.
Cheatle, A. H., King's College.
Cheetham, C. F., Manchester.
Cholmeley, W. F., St. Bartholom.
Clapham, J. T., St. Bartholomew's.
Collington, F. A., Guy's.
Cooke, M. P., Middlesex.
Cooke, T. A. B., Guy's.
Copeland, W. H. L., St. Thomas's.
Corner, H., London.
Cressy, C. J., Guy's.
Cross, E. J., St. Thomas's.
Day, F. W. H. L., University Coll.
Douty, E. H., Cambridge and Middlesex.
Dowling, E. A. G., St. Bartholom.
Dugan, F., Guy's.
Eaton, O., Manchester.
Farmer, F. R., Guy's.
Farrar, R. A., St. Bartholomew's.
Firth, J. L., King's and Univ. Coll.
Foster, M. G.. Cambridge and University College.
Franklin, L., St. George's.
Gomez, A. C., University College.
Gornall, J. P. J., Manchester.
Gott, H., Leeds.
Grant, H., Edinburgh and London.
Graves, C., St. Mary's.
Grosvenor, W. W., St. Bartholom.
Halley, W., Charing Cross.
Hanson, A. S., St. Mary's.
Hayward, C. W., Liverpool.
Heasman, W. G., St. Bartholom.
Hewer, A. E., St. Bartholomew's.
Hewlett, C. W., Guy's.
Hill, G. L., Birminghan.
Hodgetts, C. A., Toronto.
Hudson, F. H., Bristol.
Humphrey, G. H., St. Bartholom.
Hutt, C. E. St. Bartholomew's.
Johns, J. F., Leeds.
Joslen, H , Guy's.
Keller, O. E., Zurich.
Kemp, G. L., Guy's.
King, R. H., St. Bartholomew's.
LeFeuvre, W. P., Guy's.
Lewis, B. M., University College.
Locke, C. A., University College.
Liston, W. L., St. Mary's.
Mackay, P. B., St. George's.
Moreland, C. H. D., St. George's.
Metcalfe, G., Newcastle.
Miers, A., Leeds.
Nicholls, A. R., Middlesex.
Norton, H. H., St. Mary's.
Nuttall, A. E , St. Bartholomew's.
Ogle, C., St George's.
Oliver, G. H., Leeds.
Ormerod, C. E., St. Bartholomew's.
Owen, H. E., Edinburgh.
Padbury, G. J., Guy's.
Parry, A. A., Melbourne.
Pearse, A., St. Bartholomew's.
Pedley, S. E., Guy's.
Phipps, H. H., University College.
Prosser, A. B., Birmingham.
Quirk, T. A., St. Bartholomew's.
Reed, J. S., University College.
Reynolds, E. J., London.
Rigby, W. B., St Bartholomew's.
Rilot, C. F., Middlesex.
Ring, J., Middlesex.
Robertson, J., Guy's.
Rolston, T. R., Guy's.
Roberts, R. L., University College.
Scoll, T. W., St. Bartholomew's.
Shaw, J. C., St. Bartholomew's.
Slyman, W. B., St. Bartholomew's.
Spencer, T. E., St. Bartholomew's.
Smith, H. A., King's College.
Staniforth, J. W., Sheffield and St. Thomas's.
Stevens, W. E., Bristol.
Thompson, G. H , St. Bartholom.
Tunnicliffe, F. W., St. Bartholom.
Thorp, C. G., Guy's.
Tanner, E. O., University College.
Walker, A. H., Charing Cross.
Ward, W. F., St. Thomas's.
Watkins, W., St. Bartholomew's.
Wells, F. B., University College.
Williams. R. E., Guy's.
Wright, T. N., Guy's.
Young, James, Manchester.

### SOCIETY OF APOTHECARIES OF LONDON.

The following having satisfied the Court of Examiners as to their knowledge of the science and practice of medicine, surgery, and midwifery, have received certificates entitling them to practise as Licentiates of the society :—

Baly, P. P., Queen's College, Birmingham.
Burrowes, H. A , Royal Infirmary, Liverpool.
Chamberlain, E. B., London Hospital.
Lee, H. B., Sheffield and Leeds.
Mead, T. W., St. George's Hospital.
M'Leod, H. H. B., King's College.
Potter, P. de C., Manchester Infirmary.
Raynes, S. H., London Hospital.
Williams, J. C., Royal Southern Hospital, Liverpool.

**To Residents, Secretaries, and Matrons.**

The Editor will be glad to have the Regulations and each Report as issued by Asylums, Hospitals, Convalescent and Nursing Institutions, as well as those of other Charities, and an early copy in duplicate of all Appeals and Festival Notices, etc., addressed to *The Editor, The Lodge, Porchester Square, London, W.* Any superintendent, secretary, matron, or other chief official who regularly sends such information may be registered, on application to the Publisher, to receive free of cost a cover for binding The Hospital in half-yearly volumes.

**Notice to Advertisers and Subscribers.**

All Advertisements and Business Communications should be addressed to The Publisher, *not to the Editor*, The Hospital, 38, Old Jewry, E.C.
A Scale of Charges will be found in the Advertisement Sheets, page iii.

### Scraps and Gleanings.

Santiago has been afflicted with a terrible measles plague. Upwards of a thousand children died in less than two months.

Mr. Atkinson, of the Animals' Institute, will inject morphia in the event of painful accidents to horses in his neighbourhood.

Professors Von Bergmann and Gerhardt have had the significant honour of dining with the German Emperor.

A remarkable document has been issued by Mrs. Victoria Woodhull, in which she implores the Government to make it "a crime against the nation" to marry when malformed or diseased.

Mr. R. C. Scott, retired Inspector-General of Naval Hospitals, has died from the effects of inhaling nitrate of amyl.

The Italian, Succi, has concluded his thirty days' fast, at Barcelona. His health does not appear to have suffered by his long abstinence.

At the opening meeting of the Browning Society last week, a paper was read on "Paracelsus: the Luther of Medicine."

Miss Louisa McKellar has left £250 to the Metropolitan and City Police Orphanage.

The Sanitary Commissioner of Oudh says that the high rate of infant mortality in India is due to improvidence, not to unhealthy surroundings.

The Matron of Luton Cottage Hospital is being made the subject of a lively correspondence in the local papers.

There are nine individuals on the staff of Lodge Moor Hospital, Sheffield, and not a single patient for them to attend to.

In London last week the deaths registered were thirty above the average in the corresponding weeks of the last ten years, and exceeded the rate for any week since April last.

The Charing Cross Hospital Students' Dinner took place at the Holborn Restaurant on Friday last.

The possibility of the State taking a more direct part in the prevention and treatment of leprosy has recently been under the consideration of the Government of India; they have decided to do nothing at present.

The preliminary plans of the Kendray Fever Hospital have been laid before the committee.

Last year there was a decrease in the Newcastle Hospital Sunday Fund, so on Sunday last special efforts were made to recover the old ground.

The notice of a vacancy for a surgeon at Salford Hospital was only advertised three days before applications were due. The fact has aroused some indignation in local circles.

Hospital Sunday at Salisbury brought in over £200 to the funds of the infirmary and dispensary.

The Taunton Hospital wants a receiving-room, and the wards generally want making more bright and cheerful. Was there no jubilee at Taunton?

There being 13 fever patients in Maidstone Infectious Hospital, the board has refused to take in cases from the Union.

Mr. Andrew Melville has again placed the Grand Theatre, Birmingham, at the disposal of the Hospital Sunday Committee.

In Ireland the same person often holds the office of coroner and dispensing medical officer. The case of Dr. Higgins at Stradbally has just caused this fact to be commented on, as some believe that the duties of the two posts are incompatible.

The Edinburgh Dental Hospital is to be connected with the Royal Infirmary, and will move to its new quarters at Whitsuntide.

Extensive structural alterations have been completed at the Loughborough Dispensary and Infirmary, at a cost of £1,300.

It is proposed to erect a hospital at Kirkcaldy.

Marie Duncan has written an article in the *Queen* on Hospital Visiting. She insists that great tact is essential for those who would make themselves welcome as visitors to patients.

Mr. Churchill has given £100 to the Dorset County Eye Infirmary, on the condition that £300 is raised elsewhere during the present year.

At the annual meeting of the Dudley Dispensary last week, it was announced that more subscribers were needed.

The working men of Sleaford have contributed £112 to the Lincoln Hospital Fund.

The Treasurer of Leicester Infirmary acknowledges the receipt of £1,676 from the Hospital Saturday collections. Bravo, Leicester!

After a year's discussion, the Londonderry Council have decided to build a small-pox hospital.

A conversazione was held at Wisbech on October 23rd in aid of the funds of the North Cambridgeshire Hospital. The guests were received by the Mayoress (Mrs. J. T. Hiscox).

The Marquis of Hertford presided at the annual meeting of the governors of the Birmingham Orthopædic Hospital on October 26th. A deficiency of over £100 was announced, owing to a falling-off of annual subscribers.

The next meeting of The Hospitals Association will be held on Thursday, November 15th, at Eight p.m., in the Governors' Hall of St. Bartholomew's Hospital.

It is stated that the Right Honourable the Lord Mayor will preside on the 15th inst. at Barts, and that Sir Sydney Waterlow, Bart., will read a paper on "Hospital Sunday."

### Amusements and Relaxation.

### NEW PUZZLE PRIZE SCHEME.

COMMENCED OCTOBER 6th, ENDS DECEMBER 29th, 1888.

On October the 6th a New Prize Scheme was commenced in these columns. Prizes will be awarded for the best Original Puzzles in Prose or Verse, relating to any of the Social, Philanthropic, or Political topics of the day, and taking the form of Double Acrostics, Anagrams, Square Words, Decapitations, Transpositions, or any other Puzzles which the ingenuity of the competitors may suggest. (*Answers must accompany contributions.*)

*Readers* are requested to send in weekly the Number of Marks they consider each composition to be entitled to, three being the highest score for any one contribution.

Eight Prizes of Standard Books will be given, the choice of the book to be left to the winner.

First, Second, Third, Fourth, and Fifth Prizes for best Original Puzzles, of £1 1*s.*, 15*s.*, 10*s.* 6*d.*, 7*s.* 6*d.*, and 5*s.* each.

First, Second, and Third Prize for Solutions of 15*s.*, 10*s.* 6*d.*, and 5*s.* each.

N.B.—All contributions must reach the office, 38, Old Jewry, E.C., not later than the *First Post* on *Thursday* in each week.

### NOTICES TO CORRESPONDENTS.

*No competitor is allowed marks for the solution of his own puzzles. No competitor to contribute more than three puzzles at one time. No competitor will be permitted to take more than One Prize during the quarter, or more than Two Prizes in the course of the year.*

*Lady Clara.*—Observe Condition Number V.

### PUZZLES FOR SOLUTION.

**Third Week.**

No 7. Double Acrostic.

In hospitals *both* of great value are reckoned,
By the doctors the first and the nurses the second.

1. Nor animal, nor mineral, nor vegetable new;
It's neither male nor female. but something' twixt the two.
2. Oh! perfection of bliss on a hot summer's day,
And fun on a cold winter's evening to play.
3. A plant and a colour. What can it be?
Give it up? Wait till the end and you'll see.
4. An adventurous animal who thought himself nice,
Went a journey not asking his mother's advice.
5. On the weather, companions, and talk by the way.
Depends very much the success of the day. Dandy Dick.

No. 8. Original Anagram.

"I saw a grand lot meet Will." Patience.

No. 9. Triple Acrostic.

Two old opponents here you see,
As opposite as they can be;
Tho' joined by a common friend,
Of their disputes there is no end.
1. This is entirely tentative.
2. By repetition still I live.
3. To time a piquant charm I give. Lady Clara.

**Marks.**

| Names. | No. of Puzzles sent in Oct. 25th. | No. of Marks Oct. 25th. | Total of Marks. |
|---|---|---|---|
| Patience | 4 | 8 | 17 |
| Lady Clara | 5 | 13 | 19 |
| Tinie | 2 | 2 | 11 |
| Bijou | 1 | 2 | 8 |
| Thanet | 3 | 4 | 14 |
| G. D. | — | — | 3 |
| Daisy | 1 | 2 | 6 |
| Salford | — | — | 6 |
| Hercules | 3 | 3 | 9 |
| Gretta | 3 | 4 | 8 |
| Dandy Dick | — | — | 5 |
| Boadicea | 3 | 4 | 7 |
| Nipper | 5 | 7 | 7 |

**Answers.**

No. 1. Double Acrostic.

A rie L
U ndin E
T iar A
U —— V
M elpomen E
N imbu S

No. 2. Decapitation and Transposition.

Wolf, fowl, owl, low.

No. 3. Original Anagram.

The National Pension Fund for Nurses.

**Marks for Solution of Puzzles.**—October 25th.

Names.—Ruffles, 2; Daisy, 2; Lady Clara, 2; Reldas, 2; Mater, 1; Condorcet, 2; Tinie, 1; Gentian, 3; Marguerite, 3; Patience, 1; Gretta, 2; Amicus, 0.

**Conditions.**

I. Every paper sent in must be clearly written, and one side only must be used. All competitors must mark at the head of their papers the number of their competition.

II. Each paper must be signed by the author with his or her real name and address. A *nom de plume* may be added if the writer does not desire to be referred to by us by his real name. In the case of all prizewinners, however, the real name and address will be published.

III. All communications relating to prize competitions should be addressed to "The Prize Editor, The Hospital, 38, Old Jewry, London, E.C." Competitors are requested not to include in letters, etc., to the Prize Editor communications on matters of other business. All letters must be fully prepaid.

IV. The Prize Editor of The Hospital is the sole judge of the competitions, and his decision is in all cases final and without appeal.

V. The Prize Editor reserves the right to publish any paper sent in, whether it receives a prize or not. He does not hold himself responsible for any MSS. sent, nor can he undertake to return rejected competitions.

